# Reply: Cognitive behavioural therapy sessions approach ineffective for anxiety and depression in COPD: is the door closed for good?

**DOI:** 10.1183/13993003.02149-2023

**Published:** 2024-01-04

**Authors:** Stephanie J.C. Taylor, Ratna Sohanpal, Liz Steed, Karen Marshall, Moira J. Kelly, Martin Underwood, Patrick White, Hilary Pinnock

**Affiliations:** 1 Wolfson Institute of Population Health, Queen Mary University of London, London, UK; 2 Newcastle upon Tyne NHS Foundation Trust, Chest Clinic, RVI Hospital, Newcastle upon Tyne, UK; 3 Warwick CTU, Coventry, UK; 4 University Hospitals of Coventry and Warwickshire, Coventry, UK; 5 Department of Population Health, School of Life Course and Population Sciences, King's College London, London, UK; 6 Usher Institute, The University of Edinburgh, Doorway 3, Medical School, Edinburgh, UK

## Abstract

We thank A.M. Yohannes and co-workers for their generous comments on the quality of our study [1]. However, we would emphasise that our intervention was not cognitive behavioural therapy *per se*; we carefully describe our intervention as a “cognitive behavioural approach” (CBA) delivered by trained healthcare professionals. Following training, these professionals were assessed for proficiency in delivering the intervention before they were eligible to act as TANDEM facilitators and they were supervised throughout by qualified cognitive behaviour therapists, but our facilitators were not formally trained and accredited CBT therapists. However, these TANDEM facilitators were also experienced practitioners in the management of COPD and were able to support tailored self-management delivery and the holistic approach advocated by A.M. Yohannes and co-workers. This approach had the practical advantage that, if successful, it could have been rolled out within the existing NHS workforce.

## Tweetable abstract

**Our trial does not support using a CBA approach to alleviate mild/moderate anxiety and/or depression in people with moderate/severe COPD. New approaches are needed to relieve the substantial mental health burden in these patients with complex needs.** https://bit.ly/3TkkDt3

*Reply to A.M. Yohannes and co-workers*:

We thank A.M. Yohannes and co-workers for their generous comments on the quality of our study [1]. However, we would emphasise that our intervention was not cognitive behavioural therapy *per se*; we carefully describe our intervention as a “cognitive behavioural approach” (CBA) delivered by trained healthcare professionals. Following training, these professionals were assessed for proficiency in delivering the intervention before they were eligible to act as TANDEM facilitators and they were supervised throughout by qualified cognitive behaviour therapists, but our facilitators were not formally trained and accredited CBT therapists. However, these TANDEM facilitators were also experienced practitioners in the management of COPD and were able to support tailored self-management delivery and the holistic approach advocated by A.M. Yohannes and co-workers. This approach had the practical advantage that, if successful, it could have been rolled out within the existing NHS workforce.

We disagree with A.M. Yohannes and co-workers that a greater “dose” of our intervention was required. It was a pragmatic trial designed to mimic a real-life situation in which patients have varied needs. The number of sessions was tailored to the individual so that if a participant did not receive 6–8 sessions of the TANDEM intervention it was not because of a dose restriction but because of the participant: either they did not like the CBA, or they felt they had benefited as much as they could, or an exacerbation or other life events prevented further sessions. We tried to make it as easy as possible for participants to receive the intervention; the majority had the intervention in their own homes (though they had a genuine choice about where the intervention would be delivered). We offered extra sessions to those who had their intervention sessions interrupted by illness or life events (and in practice several participants had nine intervention sessions). It is hard to see how we could have delivered a greater “dose” of our intervention (or any similar cognitively behaviourally informed intervention) to our patient population, *i.e*. people with symptoms of mild to moderate anxiety and/or depression and moderate to very severe airways obstruction. Furthermore, our participants received a considerable amount of our intervention – as A.M. Yohannes and co-workers note. On average, our participants received 4.8 intervention sessions with 81% receiving two or more sessions – our minimal clinically effective dose. The minimal clinically effective dose for UK NHS Talking Therapies (formally known as IAPT, Increasing Access to Psychological Therapies) is also two sessions [2].

We agree with the conclusions of the accompanying editorial by Evans and Doe [3] that increasing referral to, and uptake of, pulmonary rehabilitation by people with COPD is paramount, but would like to clarify two misconceptions. First, the uneven randomisation ratio was intentional (to maximise power in a trial in which clustering only applied in one arm) [4], and did not arise because of the randomisation anomaly, which is fully described along with sensitivity analyses showing it did not influence our results in the accompanying supplementary materials. Second, we did conduct an internal pilot, but the role of internal pilots, as described in the seminal paper by Lancaster *et al*. [5], is to examine aspects of trial delivery, such as recruitment and trial feasibility, and not to explore intervention effectiveness, for which they are clearly underpowered. Indeed, Lancaster *et al*. [5] specifically caution against using internal pilots to look at effectiveness.

In summary, we believe that our trial has closed the door on using a CBA approach to alleviate symptoms of mild/moderate anxiety and depression in people with moderate/severe COPD. What is needed is a novel approach that can open a new door which can relieve the substantial mental health burden in these patients with complex needs.

## Shareable PDF

10.1183/13993003.02149-2023.Shareable1 This one-page PDF can be shared freely online.
Shareable PDF ERJ-02149-2023.Shareable

## References

[1] Taylor SJC, Sohanpal R, Steed L, et al. Tailored psychological intervention for anxiety or depression in COPD (TANDEM): a randomised controlled trial. Eur Respir J 2023; 62: 2300432. doi:10.1183/13993003.00432-2023 37620042 PMC10620475

[2] Clark DM. IAPT at 10: Achievements and Challenges. Date last updated: 13 February 2019. Date last accessed: 25 November 2023. www.england.nhs.uk/blog/iapt-at-10-achievements-and-challenges/

[3] Evans R, Doe G. Can the curse of mood disorders in COPD be lifted and enable pulmonary rehabilitation? Eur Respir J 2023; 62: 2301538. doi:10.1183/13993003.01538-2023 37918881

[4] Kahan BC, Rehal S, Cro S. Risk of selection bias in randomised trials. Trials 2015; 16: 405. doi:10.1186/s13063-015-0920-x 26357929 PMC4566301

[5] Lancaster GA, Dodd S, Williamson PR. Design and analysis of pilot studies: recommendations for good practice. J Eval Clin Pract 2004; 10: 307–312. doi:10.1111/j..2002.384.doc.x 15189396

