# Supplementary material for: Reply: Cognitive behavioural therapy sessions approach ineffective for anxiety and depression in COPD: is the door closed for good?
Source: Eur Respir J. 2024 Jan 4;63(1):2302149. doi: 10.1183/13993003.02149-2023 (PMC10764980; doi:10.1183/13993003.02149-2023)
Supplement: Supplementary file 1 [file ERJ-02149-2023.Shareable.pdf]

Reply: Cognitive behavioural therapy sessions approach ineffective for anxiety and depression in COPD: is the door closed for good?

Stephanie J.C. Taylor[1], Ratna Sohanpal[1], Liz Steed[1], Karen Marshall[2], Moira J. Kelly[1], Martin Underwood[3,4], Patrick White[5] and Hilary Pinnock[6]

[1]Wolfson Institute of Population Health, Queen Mary University of London, London, UK. [2]Newcastle upon Tyne NHS Foundation Trust, Chest Clinic, RVI Hospital, Newcastle upon Tyne, UK. [3]Warwick CTU, Coventry, UK. [4]University Hospitals of Coventry and Warwickshire, Coventry, UK. [5]Department of Population Health, School of Life Course and Population Sciences, King's College London, London, UK. [6]Usher Institute, The University of Edinburgh, Doorway 3, Medical School, Edinburgh, UK.

Corresponding author: Stephanie J.C. Taylor (s.j.c.taylor@qmul.ac.uk)

Shareable abstract (@ERSpublications)
Our trial does not support using a CBA approach to alleviate mild/moderate anxiety and/or depression in people with moderate/severe COPD. New approaches are needed to relieve the substantial mental health burden in these patients with complex needs. https://bit.ly/3TkkDt3

Cite this article as: Taylor SJC, Sohanpal R, Steed L, *et al*. Reply: Cognitive behavioural therapy sessions approach ineffective for anxiety and depression in COPD: is the door closed for good?. *Eur Respir J* 2024; 63: 2302149 [DOI: 10.1183/13993003.02149-2023].

This extracted version can be shared freely online.

Copyright ©The authors 2024.

This version is distributed under the terms of the Creative Commons Attribution Licence 4.0.

Received: 29 Nov 2023
Accepted: 30 Nov 2023

Reply to A.M. Yohannes and co-workers:

We thank A.M. Yohannes and co-workers for their generous comments on the quality of our study [1]. However, we would emphasise that our intervention was not cognitive behavioural therapy *per se*; we carefully describe our intervention as a "cognitive behavioural approach" (CBA) delivered by trained healthcare professionals. Following training, these professionals were assessed for proficiency in delivering the intervention before they were eligible to act as TANDEM facilitators and they were supervised throughout by qualified cognitive behaviour therapists, but our facilitators were not formally trained and accredited CBT therapists. However, these TANDEM facilitators were also experienced practitioners in the management of COPD and were able to support tailored self-management delivery and the holistic approach advocated by A.M. Yohannes and co-workers. This approach had the practical advantage that, if successful, it could have been rolled out within the existing NHS workforce.
